# Chondromatosis of the temporomandibular joint as a cause of reflex otalgia

**DOI:** 10.1016/j.bjorl.2023.101284

**Published:** 2023-06-29

**Authors:** Marcelo Augusto Cini, Mariana Demarchi Avelino, João Guilherme Lacy Araújo Machado, Ricardo Lorota de Resende Junior, Alan dos Santos

**Affiliations:** Universidade do Oeste Paulista (UNOESTE), Guarujá, SP, Brazil

**Keywords:** Chondromatosis, Otalgia, Temporomandibular disorder

## Introduction

Temporomandibular Disorder (TMD) is a disease according to the International Statistical Classification of Diseases a significant public health problem, which is considered the most common cause of chronic pain of non-dental origin.^1^

In this regard due to the anatomical proximity between the ear and the TMJ, many patients report an otological complaint which may be associated with a type of TMD including tinnitus, auricular fullness, vertigo, itching, joint noise sensation and otalgia; therefore, the first evaluation of the otolaryngologist is common,^2^ followed by oral and maxillofacial evaluation or a dental surgeon specialized in orofacial pain.

Synovial Chondromatosis (CS), more common in larger joints such as knee and shoulder andrarelyin the TMJ.^3^ This pathology is characterized by the proliferation of synovial tissue with the production of cartilaginous nodules that are located in the joint spaces, usually the superior one, but may involve the inferior compartment^4^ and may be attached or untied in the joint,^6^ and in more advanced cases, may proliferate to extra-articular spaces.^5^

Thus, the objective of this study is to report a rare case of CS in the TMJ with a complaint of related otological symptoms and with the success through arthroscopic surgical treatment.

## Case report

This study was conducted within ethical criteria and approved by the Research Ethics Committee of the Universidade do Oeste Paulista ‒ UNOESTE of Presidente Prudente, Brazil with protocol number 7770.

Patient M.C.R., 37-years old, male, Caucasian, presented with a complaint of pain in the left ear for one year. At the beginning of the symptom, he sought an otolaryngologist who performed all the investigation through clinical and complementary exams, without systemic changes and with otoscopy showing a slightly convex, translucent, mobile, and intact tympanic membrane. He came to the conclusion that the otalgic complaint was not justified, and the patient was forwarded for evaluation by a professional dentist specializing in Temporomandibular Disorders and Orofacial Pain.

Through the propaedeutic of clinical examination without obtaining an image, Temporomandibular Disorder (TMD) was diagnosed as the cause of the symptom and conservative clinical treatment was performed for one year at the expense of physiotherapy sessions, photobiomodulation with low intensity laser, electrotherapy, interocclusal devices and occlusal adjustments. However, due to no improvement in the complaint, the patient was forwarded for evaluation by the dental specialty of Oral and Maxillofacial Surgery.

**Figure 1:**
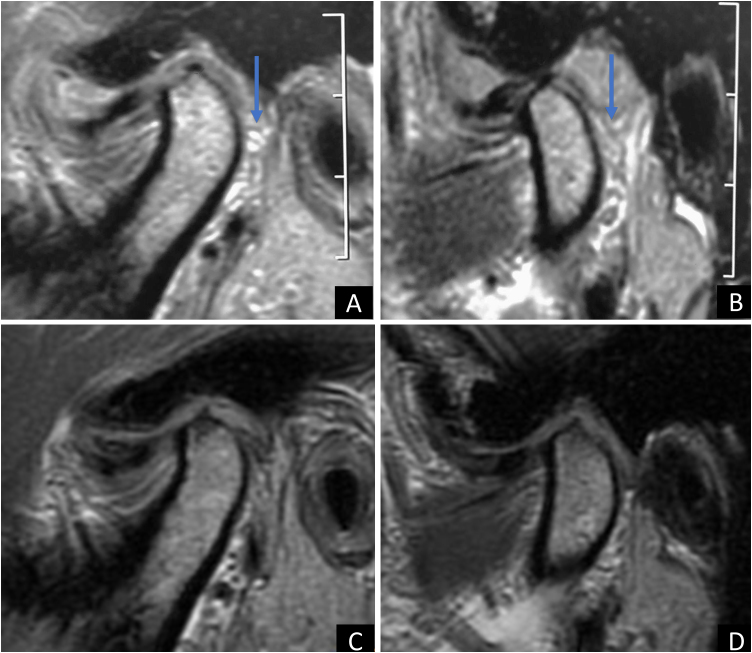
TMJ MRI. In A, mouth closed and B mouth open in the preoperative period. In C, mouth closed and D mouth open within 6-months of surgery.

Afterwards clinical examination and complementary imaging (magnetic resonance imaging) it was possible to observe the presence of numerous free bodies (synovial chondromatosis) in the left Temporomandibular Joint (TMJ) (Fig. 1A and B). The patient had never had joint complaints previously to the reported episode, had no general health problems and was not using any continuous medication, only symptomatic medications for the complaint.

The loco regional examination showed arthralgia on palpation in the left TMJ region with radiating to the left ear, without the presence of noises or other relevant signs.

**Figure 2:**
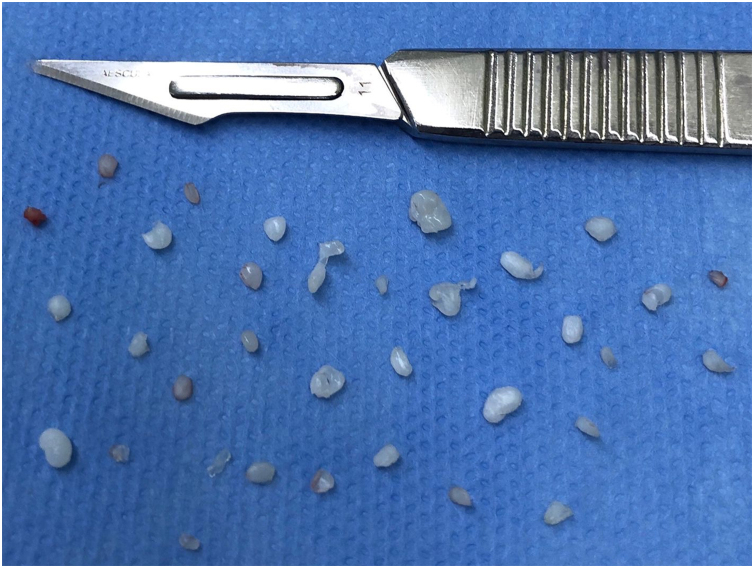
Free bodies of TMJ synovial chondromatosis removed during arthroscopic surgery.

**Figure 3:**
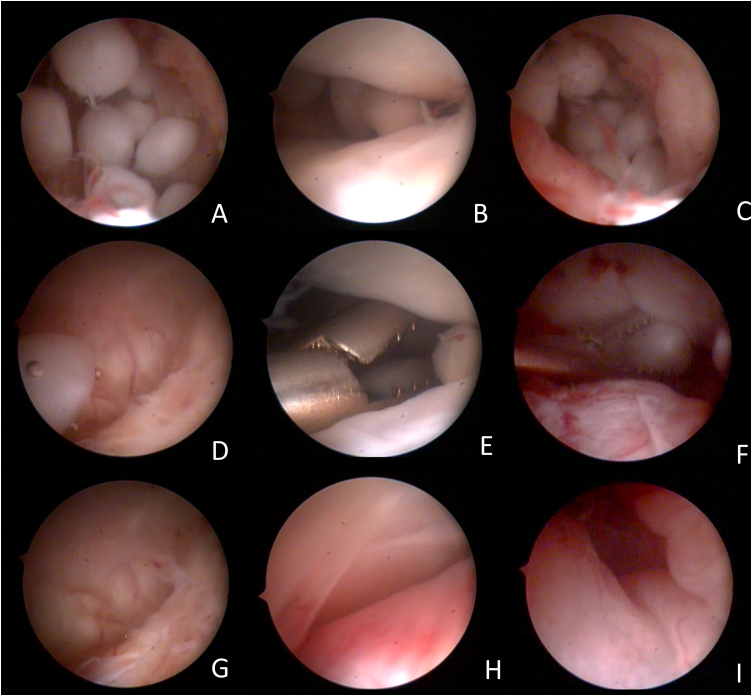
Arthroscopic images of the surgical procedure. (A) free bodies in the retrodiscal region; (B) free bodies in the middle region of the upper TMJ compartment; (C) free bodies in the posterior medial recess of the TMJ; (D) free body in the anterior recess of the anterior compartment of the TMJ, coming from the posterior region by the flow of lysis and washing; (E) pinching of free bodies in the middle region of the upper TMJ compartment; (F) free-body clamping in the posterior medial recess of the TMJ; (G) free anterior recess after removal of free bodies; (H) Middle region of the upper compartment of the free TMJ after removal of free bodies; (I) Free retrodiscal region after removal of free bodies.

The patient underwent arthroscopic surgery on the left TMJ with the aim of removing free bodies. Thirty-four free bodies were found and removed (Fig. 2), mostly in the region of the retrodiscal zone of the upper TMJ compartment (Fig. 3). In the immediate postoperative period, the patient no longer had any complaints, and 6-months after the operation, a new magnetic resonance examination was requested, where the presence of free bodies was not observed (Fig. 1C and D).

## Discussion

Chondromatosis affecting the temporomandibular joint is considered a rare event.^6^ Due to this fact, it is common to delay the correct diagnosis of TMJ synovial chondromatosis, according to a recent review, the duration of symptoms until diagnosis seems to be high about 80% of cases with duration of symptoms until diagnosis on average of 2-years, corroborating the data of our present clinical case.

Particularly in the case reported, an important point that we must discuss is that the patient's symptom was isolated, the reflex otalgia, did not present crackling, limitation or deviation in mouth opening, common signs reported in the literature and presented in TMD and CS. Therefore, what we discussed here is that even without common complaints of internal TMJ disorders, CS can cause restricted otological complaints, however, arising from a joint pathology, even without presenting signs and symptoms common to temporomandibular disorders. A possible explanation for this fact is that the greatest amount of chondromatosis free bodies was present in the retrodiscal region, a region of great innervation and TMJ sensitivity, causing this patient's reflex otalgia.

The recommended treatment method for CS is surgical^3^, ^7^, ^8^, ^9^ Currently, some authors advocate a less invasive technique, such as arthroscopic surgery,^9^ however, invasive treatment using open TMJ surgery is the most cited in the cases reported in the literature.^7^ Open surgery remains valid as a form of treatment up to the present-day, however several technologies have emerged in recent years and are also being applied to the treatment of temporomandibular disorders.

Currently, arthroscopic surgery for the surgical treatment of TMJ CS has been gaining ground due to the positive results with the technique. Cai et al.^8^ demonstrated good results in a series of 33 cases treated with arthroscopic surgery with a follow-up of 38 months without cases of recurrence, noting that the advantages of this technique would be less invisibility, shorter surgery time, less scarring and less chance of facial nerve injury.

An important aspect of arthroscopic surgical treatment is the power of navigating inaccessible spaces in open surgery. Bai et al.^10^ suggest starting with open surgery and complementing with arthroscopic surgery to reach spaces, such as posterior and anterior recesses, which are difficult to visualize in open surgery. In this study, hidden free bodies were observed in the medial sulcus of 14 patients (located in the anterior recess of eight patients and in the region of the posterior recess of six patients) of the 36 patients who underwent first open surgery followed by arthroscopy in the study. In our case, we observed the largest amount of free bodies in the medial region of the retrodiscal zone, however, with the flow of lysis and washing, we observed the transfer of free bodies to other spaces of the upper compartment, therefore a careful browsing is necessary in all areas of this region.

Currently, studies seem to suggest arthroscopic surgery when the CS of the TMJ is restricted to the upper intra-articular space and open surgery when the CS embrace the extra-articular space.^3^, ^11^, ^12^, ^13^, ^14^ However, even when the surgery is open, the use of an arthroscope is suggested for browsing the medial regions of the joint capsule.^13^

## Conclusion

Despite the TMJ synovial chondromatosis being rare, it is important in the presence of otalgia, especially in cases of non-involvement of otological pathologies, or when considering TMD's, consider it as a differential diagnosis combined with MRI as a complementary exam and decisive for reducing the time for diagnosis. Finally, in cases of intra-articular CS, arthroscopic surgery is a viable, less invasive, and efficient option in the treatment of TMJ CS.

## Conflicts of interest

The authors declare no conflicts of interest.
